# Enhancement of DNA hypomethylation alterations by gastric and bile acids promotes chromosomal instability in Barrett’s epithelial cell line

**DOI:** 10.1038/s41598-022-25279-y

**Published:** 2022-12-01

**Authors:** Iku Abe, Koichi Suzuki, Yasuaki Kimura, Sawako Tamaki, Yuhei Endo, Kosuke Ichida, Yuta Muto, Fumiaki Watanabe, Masaaki Saito, Fumio Konishi, Toshiki Rikiyama

**Affiliations:** 1 Department of Surgery, Saitama Medical Center, Jichi Medical University, 1-847, Amanuma-Cho, Omiya-Ku, Saitama, 330-8503 Japan; 2 Department of Surgery, Nerima-Hikarigaoka Hospital, 2-5-1, Hikarigaoka, Nerima-ku, Tokyo, 179-0072 Japan

**Keywords:** Cancer, Gastrointestinal cancer, Gastric cancer

## Abstract

Gastric and bile acid reflux leads to chronic inflammation, resulting in methylation alterations in Barrett’s esophagus (BE) together with chromosomal instability (CIN). We investigated DNA hypomethylation following acid exposure and confirmed its significance in BE-related carcinogenesis by inducing CIN in vitro. OACP4C, an esophageal cancer cell line, and CP-A, a non-dysplastic cell line originating from BE, were exposed to acidic conditions using deoxycholic acid. CP-A exhibited substantially increased DNA hypomethylation of alpha satellite sequences in the centromere region, as well as increased levels of alpha satellite transcripts, but no changes were observed in the long interspersed nucleotide element-1 sequences distributed throughout the entire genome. These changes were not clearly found in OACP4C. Copy number changes at specific chromosomes were identified in CP-A, along with an increased number of cells exhibiting abnormal segregations, whereas these changes were rarely observed in OACP4C. The changes were maintained after several cell divisions. These findings suggest that alpha satellites are likely targets of DNA hypomethylation induced by acid exposure. CP-A was more sensitive to acid exposure than OACP4C, indicating that acid-induced DNA hypomethylation is involved in cancer development rather than progression, which could be involved in the underlying mechanism of esophagogastric junction carcinoma development.

## Introduction

Gastric cancer is one of the most common causes of cancer death^1^. Helicobacter pylori (H. pylori) infection has been reportedly involved in carcinogenesis by inducing chronic inflammation and stimulating aberrant DNA methylation^2,3^. Therefore, H. pylori is regarded as an important risk factor for gastric cancer. In a prospective study, eradication of H. pylori after endoscopic treatment for early gastric cancer decreased metachronous multiple gastric cancers by one-third^4^. Subsequently, widespread H. pylori eradication, combined with improved hygiene reduced the incidence of gastric cancer in recent years^5^.

In contrast, esophagogastric junction (EGJ) carcinoma has been increasing worldwide, particularly in Western countries^6,7^. Various risk factors for EGJ carcinoma have been reported, such as smoking^8^, obesity^9,10^, sex^11^, and gastroesophageal reflux disease (GERD)^12,13^. Among these, GERD is strongly implicated in EGJ carcinogenesis^14^. GERD leads to extensive exposure to gastric and bile acids, resulting in chronic inflammation and oxidative stress followed by DNA damage^15^, which is believed to trigger EGJ carcinogenesis^16^. In chronic inflammation, the squamous epithelium of the esophageal mucosa is replaced by columnar epithelium, which is called Barrett's esophagus (BE), forming a precancerous lesion of EGJ cancer^17^. BE progresses to metaplasia, dysplasia and cancer^13,18^. In patients with severe esophageal mucosal damage or BE, aspirate fluids from the EGJ lesion possess a higher concentration of bile acid and consequent lower pH^19^, whereby the absence of H. pylori infection alters the gastric environment making it more acidic and enhancing its susceptibility to carcinogenesis.

Epigenetic alterations are also induced by chronic inflammation, such as aberrant DNA methylation of CpG islands in the promoter region, leading to transcriptional repression of tumor suppressor genes^20–22^, which are involved in the multistep carcinogenesis of BE^23^. Several genes are reported to be methylated in BE, such as APC, ESR1, CDH1 and CDKN2A (also known as p16INK4a)^24,25^. Epigenetic alterations that occur in esophageal adenocarcinoma (EAC) are already present in BE^26,27^.

Genome-wide DNA hypomethylation is another type of epigenetic alteration that is frequently observed in repetitive sequences that are normally methylated; therefore, they are ideal targets for DNA hypomethylation^28–30^. We have focused on satellite DNA, comprising highly repetitive noncoding sequences located in centromere regions^30,31^ and have reported that the accumulation of DNA hypomethylation at the satellite DNA in normal gastric tissues enhances susceptibility to gastric cancer along with H. pylori infection^32^. The appropriate transcription of satellite DNA is essential for accurate chromosomal segregation^33^. The extent of hypomethylation in satellite DNA is associated with the levels of satellite alpha transcript (SAT), which is transcribed from satellite DNA^30^. Overexpression of satellite DNA has been found in various cancer tissues, suggesting its involvement in carcinogenesis^34^. We have reported that overexpression of SAT induced by a lentivirus-expressing vector leads to a change in the copy number at a specific chromosome^30^.

Chromosomal instability (CIN) is a major driver of tumor evolution and a hallmark of cancer, and results from ongoing errors in chromosome segregation during mitosis. Experimental studies using mouse models have provided evidence that CIN is induced by global hypomethylation, resulting in cancer^28,29^. Barrett's esophageal carcinoma originating from BE has been reported to be associated with CIN, such as loss of heterozygosity (LOH)^35^ and chromosomal copy number alterations in clinical specimens^36^. In addition, a recent genomic analysis from The Cancer Genome Atlas (TCGA) revealed that EGJ carcinoma was characterized as a feature of CIN based on molecular subtypes^37^. However, evidence of a direct connection between CIN and acid exposure in BE has not been reported.

In this study, we aimed to elucidate whether chronic inflammation caused by gastric and bile acids induces epigenetic alterations and leads to CIN in Barrett’s epithelial cell lines, which could be a potential mechanism underlying the development of EGJ carcinoma.

## Results

### Viability of OACP4C and CP-A cell lines exposed to acid and deoxycholic acid

**Figure 1:**
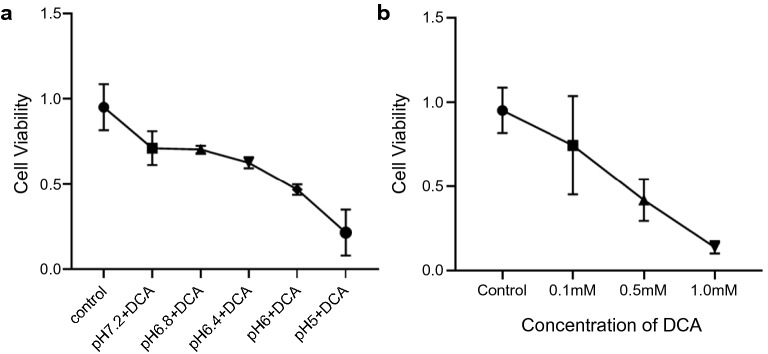
Cell viability of the OACP4C cell line under exposure of acids and deoxycholic acid. Cell viability of OACP4C cells treated at several pHs in combination with 100 μM deoxycholic acid (DCA) (**a**), and with several doses of DCA for 1 h (**b**). Approximately 50% cell viability was maintained in cell lines treated with acid at pH ≥ 6.4 in combination with 0.1 mM DCA.

First, we assessed cell viabilities of OACP4C and CP-A cell lines exposed to acids and deoxycholic acid (DCA). After the cells grew subconfluently, two and three exposures to acid and DCA were performed in OACP4C and CP-A cell lines, respectively. Cell viability gradually declined in a dose-dependent manner in both cell lines. Approximately 50% cell viability was maintained in cell lines treated with acid at pH ≥ 6.4, in combination with 0.1 mM DCA (Fig. 1).

### Relative hypomethylation levels of alpha satellite and long interspersed nucleotide element-1 repetitive sequences on exposure to acid and deoxycholic acid in cell lines

**Figure 2:**
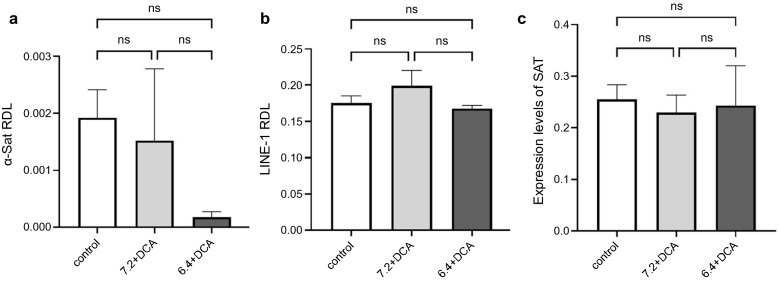
Relative hypomethylation levels of alpha satellite and LINE-1 repetitive sequences and expression levels of satellite alpha transcripts when exposed to acid and deoxycholic acid in OACP4C cells. To assess the relative hypomethylation levels, the relative demethylation levels (RDL) of alpha satellite (α-Sat) and LINE-1 repetitive sequences were determined. The expression of satellite alpha transcripts (SAT) was also calculated. There was no significant difference in α-Sat, LINE-1 RDL and expression levels of SAT under several conditions of acid deoxycholic acid in OACP4C cells, respectively. *ns*; no significance.

**Figure 3:**
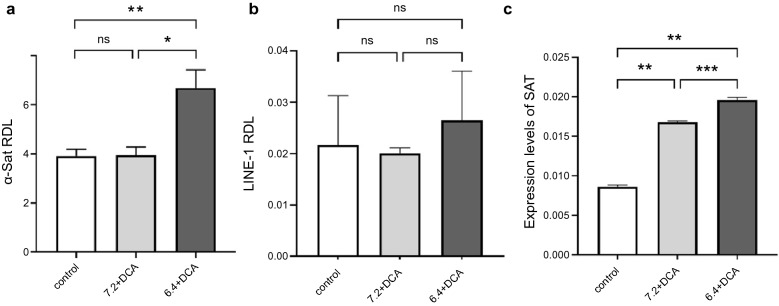
Relative hypomethylation levels of alpha satellite and LINE-1 repetitive sequences and expression levels of satellite alpha transcripts in response to acid exposure and deoxycholic acid in CP-A cells. The levels of alpha satellite (α-Sat) were substantially increased in response to acid exposure in CP-A (**a**) although there were no changes in those of LINE-1 RDL (**b**). The expression levels of SAT were significantly elevated based on the extent of DNA hypomethylation in response to acid exposure (**c**). *ns*; no significance *p = 0.0001, **p < 0.0001, ***p = 0.0029.

We explored the changes in DNA hypomethylation levels of alpha satellite (α-Sat) and long interspersed nucleotide element-1 (LINE-1) repetitive sequences in OACP4C and CP-A cell lines after exposure to acid and DCA. α-Sat and LINE-1 are repetitive sequences located in the centromeric region and distributed throughout the entire genome, respectively. To assess DNA hypomethylation levels, the relative demethylation levels (RDL) of α-Sat and LINE-1 were determined after several passages of cell lines exposed to various acidic conditions. In addition, the expression of satellite alpha transcripts (SAT), non-cording RNA transcribed from α-Sat, was measured using RT-qPCR. No significant changes in the levels of either α-Sat or LINE-1 RDLs were observed after exposure to acid and DCA in OACP4C (Fig. 2a,b). The expression levels of SAT were not altered by exposure to acid or DCA (Fig. 2c). In addition, another human esophageal adenocarcinoma cell line, OACM5.1C also exhibited no significant differences in α-Sat RDL, LINE-1 RDL and expression levels of SAT under several conditions of acid and DCA (Supplementary Fig. S1). Conversely, the levels of α-Sat RDL significantly increased in response to acid exposure in CP-A (3.91 ± 0.237, 3.95 ± 0.275 and 6.68 ± 0.678 in control, 7.2 + DCA, and 6.4 + DCA, respectively, *p = 0.0001, **p < 0.0001, Fig. 3a), despite the absence of changes in the levels of LINE-1 RDL (Fig. 3b). In addition, the expression levels of SAT were significantly elevated in response to acid exposure (0.008 ± 0.001, 0.017 ± 0.001, and 0.020 ± 0.001 in control, 7.2 + DCA, and 6.4 + DCA, respectively; **p < 0.0001, ***p = 0.0029, Fig. 3c), suggesting that α-Sat repetitive sequences in the centromere region are likely targets for DNA hypomethylation induced by acid exposure, rather than LINE-1 repetitive sequences distributed throughout the entire genome. Comparing the response to acid exposure of three cell lines, CP-A, a non-dysplastic cell line, was more sensitive to acid exposure than OACP4C and OACM5.1C, cancer cell lines. These data imply that exposure to acid and bile acid led to aberrant hypomethylation of α-Sat repetitive sequences and aberrant expression of SAT in the centromere region. Furthermore, CP-A is more susceptible to these abnormalities than OACP4C and OACM5.1C cells, and could be involved in the carcinogenesis of the EGJ originating from BE.

### Copy number changes in CP-A, OACP4C and OACM5.1C cell lines after exposure to acid and deoxycholic acid in cell lines

**Figure 4:**
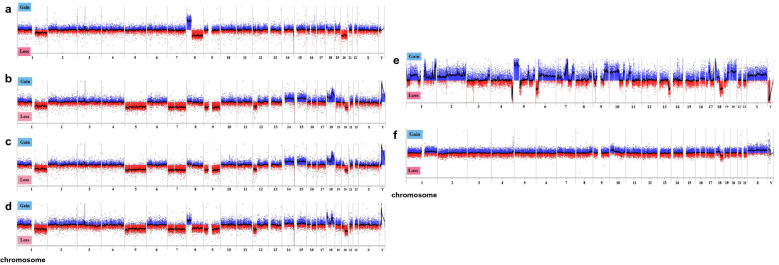
Copy number changes in CP-A and OACP4C cell lines when exposed to acid and deoxycholic acid in cell lines. Copy number changes were explored using microarray-based comparative genomic hybridization (array CGH). Representative copy number alterations identified in CP-A (**a–d**) and OACP4C (**e**,**f**). Copy number alterations before treatment with acid and DCA (**a**,**e**) and when treated with acid and DCA (**b**–**d**,**f**). Genomic DNA from each cell line without acid or DCA was labeled with Cy5-dUTP and compared to a Human Male Reference DNA (Agilent), labeled with Cy3-dUTP (i.e., naïve CP-A vs reference). Genomic DNA of each cell line treated with acid and DCA was labeled with Cy5-dUTP and compared to that of corresponding cell line treated without acid or DCA, which was labeled with Cy3-dUTP (i.e., CP-A treated with acid and DCA vs naïve CP-A). Copy number alterations at specific chromosomes were observed in CP-A and they were identical at different passage numbers (**b**–**d**) whereas these changes were rarely seen in OACP4C before and after treatment with acid and DCA (**f**). Gains are shown as blue lines in the upper area and losses are red lines in the lower area, respectively. Numbers represent the chromosome number. In each chromosome area, the short arm is located on the left side, and the long arm is on the right side.

In our previous study, we reported that SAT overexpression led to a change in copy number at specific chromosomes with mitotic errors in human mammary epithelial cells^30^. Therefore, we investigated changes in copy number alterations of chromosomes in CP-A under increased SAT expression induced by DNA hypomethylation compared to OACP4C and OACM5.1C. Microarray-based comparative genomic hybridization (array CGH) analysis revealed that copy number alterations increased in CP-A after treatment with acid and DCA. Before treatment with acid and DCA, CP-A originally harbored copy number alterations in chromosomes 1, 8 and 20 (Fig. 4a). Copy number alterations in CP-A increased after treatment with acid and DCA in specific chromosomes, such as the loss of chromosomes 5, 7, 8, 9, and 12, and the gain of chromosomes 3, 10, 18, 20, and Y (Fig. 4b–d). In contrast, OACP4C and OACM5.1C originally harbored copy number alterations before treatment (Fig. 4e and Supplementary Fig. S2a, respectively) but they remained unchanged before and after treatment with acid and DCA (Fig. 4f and Supplementary Fig. S2b). These results are in agreement with the changes in DNA hypomethylation and SAT expression levels under acid exposure. DNA hypomethylation in α-Sat RDL and expression of SAT were enhanced by acid exposure in CP-A (Fig. 3) but not in OACP4C (Fig. 2) or OACM5.1C (Supplementary Fig. S1). Copy number alterations induced by acid exposure were maintained after several passages of the cell lines (Fig. 4b–d), indicating that acid-induced CIN is inherited during chromosome replication. Taken together, our data suggest that exposure to acid and bile acid leads to CIN in CP-A by the induction of DNA hypomethylation of α-Sat followed by aberrant expression of SAT.

### Abnormal segregation in CP-A cell line under induction of DNA hypomethylation of α-Sat and aberrant expression of SAT

**Figure 5:**
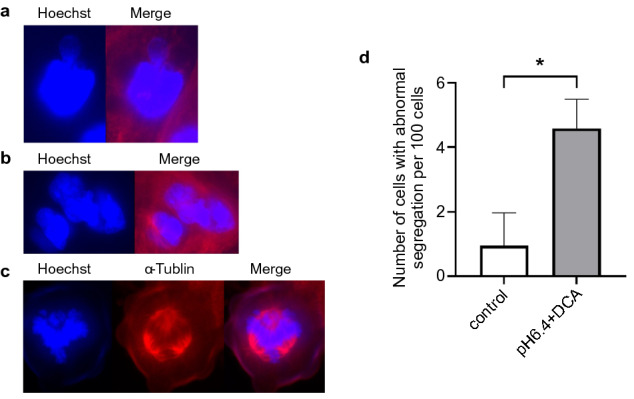
Mitotic abnormalities and number of cells with abnormal segregation in CP-A cell line under induction of DNA hypomethylation of α-Sat. The images represent findings suggestive of mitotic abnormalities such as micronuclei (**a**), and multiple nuclei (**b**), and abnormal segregation (**c**). The number of cells with mitotic abnormalities increased considerably on treatment with acid and bile acid (pH 6.4 + DCA) compared to those without treatment (**d**). The incidence of mitotic abnormalities was evaluated in 100 cells per field and average of 6 fields which was substantially greater in cells with lower pH and DCA. *p < 0.05.

Abnormal segregation of chromosomes was also induced by SAT overexpression in our previous study^30^. Therefore, we confirmed that abnormal segregations were detected in CP-A by the induction of DNA hypomethylation of α-Sat and aberrant expression of SAT. CP-A cells exhibited abnormal division, including micronuclei, multiple nuclei, and abnormal segregation (Fig. 5a–c). The number of cells with abnormal segregations increased considerably by treatment with acid and bile acid (pH 6.4 + DCA) compared to those without treatment (Fig. 5d). OACP4C originally exhibited mitotic abnormalities, but the number of cells with these abnormalities was not increased with acid exposure (data not shown).

### Differences in gene expression in CP-A cells treated with acid and deoxycholic acid and those without acid

OF the total 21,453 genes, 1418 genes were identified as differentially expressed by more than 2.0-fold between the samples. Among the genes that showed considerable differences, 573 were upregulated and 844 were downregulated. Gene annotation and pathway ontology analyses were performed using the Database for Annotation, Visualization, and Integrated Discovery (DAVID) and Kyoto Encyclopedia of Genes and Genomes (KEGG), which identified 10 pathways in 573 upregulated genes (Supplementary Table S1) and 5 pathways in 844 downregulated genes (Supplementary Table S2). The upregulated pathways were unlikely to be associated with the cell cycle or cancer-related pathways, whereas the downregulated pathways were likely linked to the cell cycle and cell division pathways.

### Methylation microarrays

**Figure 6:**
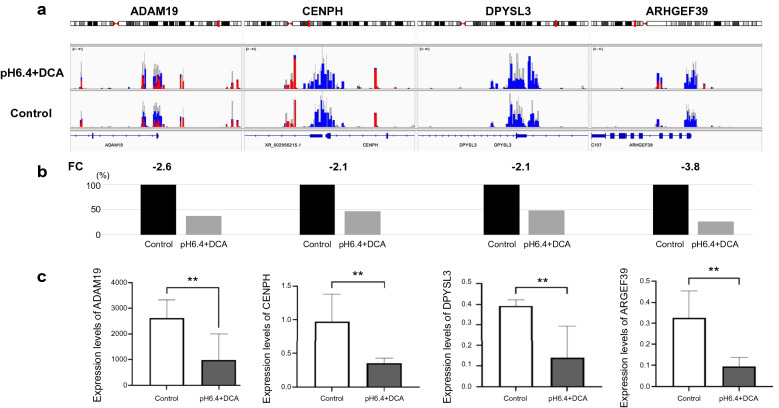
Change in DNA methylation levels at the promoter regions of representative downregulated genes determined using mRNA gene expression arrays and RT-qPCR assays for CP-A cells. (**a**) No change in DNA methylation at the promoter regions in these 4 genes, in which gene expression was downregulated after exposure to acid and DCA in CP-A cells. The promoter region of each gene is shown in red for DNA methylation and blue for demethylation. The promoter region is visualized as a blue box above the gene name. (**b**) Fold-changes of gene expression in these 4 genes determined using mRNA gene expression arrays in CP-A cells with PH6.4 + DCA were seen, when compared with that in CP-A cells without acid and DCA exposure. The percentage of gene expression in control cells was modified to 100%. FC; fold-change of gene expression between PH6.4 + DCA and control. (**c**) The expression levels of these 4 genes determined by RT-qPCR assays were significantly decreased in CP-A cells exposed to pH 6.4 + DCA compared to controls. The statistical significance between groups was determined using a two-tailed Student’s *t*-test. **p < 0.05.

To verify whether alterations of gene expression were induced by copy number changes or DNA methylation at the promoter regions of genes, we performed methylation microarrays for CP-A cells. Owing to the loss of chromosome in chromosome 5, there were some downregulated genes without alterations of DNA methylation at the promoter regions. Methylation and gene expression microarrays revealed that there were some downregulated genes without a change in the levels of DNA methylation at their promoter regions before and after exposure to acid and bile acid. Figure 6a shows representative methylation levels at promoter regions of four genes, *ADAM19*, *CENPH*, and *DPYSL3* located on chromosome 5, and *ARHGEF39* located on chromosome 9. Copy number of these chromosomes is lost in CP-A treated with acid and DCA. These four genes showed no change in methylation levels at their promoter regions after treatment with acid and DCA (Fig. 6a). Table 1 shows comparison of methylation rates of the four genes between before and after treatment with acid and DCA in CP-A. No significant difference in coverage and the ratio of methylation was seen in each gene between before and after treatment with acid and DCA. The expression levels of these genes were, however, downregulated, indicating these changes in gene expression were owed to the loss of chromosomes. Fold-changes of gene expression, determined using mRNA gene expression arrays, are shown in Fig. 6b and the decreased levels of gene expression determined by RT-qPCR assays are displayed in Fig. 6c, with statistical significance (p < 0.05). In addition, there were also other downregulated genes with increased levels of methylation at their promoter regions, suggesting that the suppression of gene expression was involved in DNA methylation in concert with the loss of chromosomes.

**Table 1:** Comparison of methylation rates of the four genes between before and after treatment with acid and DCA in CP-A.

Gene name	Chromosome	CP-A Control		CP-A pH6.4 + DCA		P-value
		Promoter coverage	Promoter fraction methylated	Promoter coverage	Promoter fraction methylated	
ADAM19	chr5	994	0.370221328	741	0.556005398	ns
CENPH	chr5	1024	0.139648438	662	0.181268882	ns
DPYSL3	chr5	1145	0.052401747	793	0.137452711	ns
ARHGEF39	chr9	496	0.002016129	429	0.004662005	ns

### Comparison of relative hypomethylation levels of alpha satellites in clinical specimens from 14 patients with EGJ cancer

**Figure 7:**
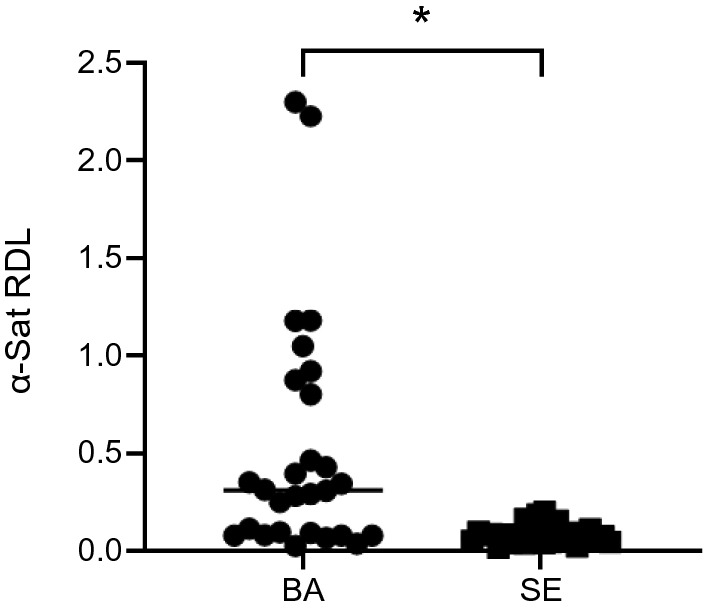
Comparison of the relative hypomethylation levels of alpha satellite between tumor tissues and squamous epitheliums in patients with Barrett's adenocarcinoma. The α-Sat RDL in Barrett's esophageal adenocarcinoma (BA) and normal squamous epithelial mucosa (SE) were compared. Plot of α-Sat RDL for each case in each group, median values are shown as horizontal bars. BA had significantly higher α-Sat RDL compared to SE. The statistical significance between groups was determined using the non-parametric Mann–Whitney U test. *p < 0.05.

To explore any closed connections between Barrett's adenocarcinoma and acid-induced hypomethylation in clinical specimens, the relative hypomethylation levels of α-Sat were compared between tumor tissues and squamous epitheliums from 14 patients with Barrett's adenocarcinoma. The α-Sat RDL of tumor tissues was higher than that of squamous epitheliums, suggesting acid-induced hypomethylation is involved in the development of Barrett's adenocarcinoma (0.3139 in tumor tissues and 0.06631 in squamous epitheliums, respectively, Fig. 7).

## Discussion

In this study, we demonstrated that exposure to acid and bile acid enhances DNA hypomethylation alterations at α-Sat repetitive sequences in the centromere region, resulting in increased expression of non-coding RNA transcribed from α-Sat sequences (SAT). Consequently, induction of DNA hypomethylation and aberrant SAT expression led to CIN with mitotic errors in a Barrett’s epithelial cell line. These changes were identical after several passages of the cell line. Our findings exhibited, for the first time, a direct connection between acid exposure and CIN in Barrett’s epithelial cell line, which could be involved in a mechanism underlying the development of EGJ carcinoma originating from BE.

Esophageal samples from patients with BE mainly consists of bile salts at low or neutral pH^38^. The combination of bile salts and acid is, therefore, widely used in in vitro models. Bile salts consist of primary bile acids, which are those synthesized by the liver, and secondary bile acids which result from bacterial actions in the colon. Secondary bile acids including deoxycholic acid (DCA) and lithocholic acid (LCA) are higher in patients with BE, compared to GERD patients and controls^38^. In addition, conjugated bile acids are higher in patients with BE, which leads to toxic properties in connection with the development of BE.

Most bile acids exist in their soluble, unionized form at pH 3–6, allowing them to enter epithelial cells and affect important pathways^39^. At lower pH, bile acids are precipitated and no longer damaging to the epithelium, while at higher pH, bile acids exist in their non-damaging ionized form^40^ Jenkins, G. J. et al. demonstrated that the effect of acidity on DCA-induced cytotoxicity in an esophageal adenocarcinoma cell line, OE33 for 24 h with DCA (100–400 mM) at pH 7.4 and 6. DCA at neutral pH (pH 7) also induced the release of reactive oxygen species (ROS) and micronucleus^41^. In our study, cell viability was extremely low in acidic condition of pH 6 or lower, after 1 h of exposure. Therefore, we adopted pH 6.4 as the most acidic condition under which half of the cells could survive.

Various incubation periods have been reported, ranging from 1 min to up to 48 h^42^. A short period of approximately 3 min is most comparable to the reflux episodes in vivo based on 24-h pH monitoring^43^. However, incubation periods need to be longer to obtain comparable results in vitro because reflux episodes are identified by pH monitoring in negative controls who are absent of clinical symptoms with refluxate^38,44^. Based on the successful induction of cytotoxicity in vitro with long incubation periods, such as exposure with DCA for 24 h reported by Jenkins et al. and chenodeoxycholic acid (CDCA) for 48 h by Soma et al., cell lines were treated for 48 h in our study. Pulsed bile acid treatment has been reported to induce proliferation, but continuous exposure led to suppression of proliferation^16^, which is consistent with our data that half of the cells did not survive. Jenkins et al. reported that continuous exposure with DCA for 24 h induced the release of ROS and micronucleus under suppression of cell proliferation.

Induction of DNA hypermethylation at the promoter region has been well described in connection with esophageal carcinogenesis under extensive exposure to acid and bile acid in clinical specimens^21,24,45,46^ and in vitro^23^. However, few investigators have addressed DNA hypomethylation. Bajpai et al. reported that DNA hypomethylation was recognized in gene bodies, intergenic regions, repetitive elements, CpG islands, and shores on exposure to acid and bile acid^47^. We have previously addressed hypomethylation in connection with chromosomal stability in gastrointestinal tumors^32,48,49^.

Repetitive DNA sequences are globally distributed in the human genome^50^ and are ideal targets for DNA hypomethylation^28–30^. In this study, hypomethylation levels were evaluated using LINE-1 and α-Sat repetitive sequences. α-Sat RDL increased considerably in response to acid exposure in CP-A, despite no changes occurring in LINE-1 RDL. Centromeric repeats are likely hypomethylated compared with repetitive sequences throughout the entire genome. There are several reports associating centromeric repeats with targets of DNA hypomethylation in connection with chromosomal instability. Amir Eden et al. demonstrate that LOH rate in hypomethylated cells is the result of a specific effect of hypomethylation on the stability of centromeric or pericentric regions^28^. Francois Gaudet et al. reported that reducing hypomorphic DNA methyltransferase 1 expression to 10% of wild-type levels, in mice, results in substantial genome-wide hypomethylation at centromeric repeats with a high frequency of chromosome 15 trisomy^29^. These results support our data, suggesting that α-Sat repetitive sequences in the centromere region are the likely targets for DNA hypomethylation induced by acid exposure in connection with chromosomal instability. Indeed, we did not verify whether LINE-1 loci may be hypomethylated but the effect is diluted due to their abundance. Furthermore, acid-induced hypomethylation was observed in a non-dysplastic cell line, CP-A, but not in the human esophageal adenocarcinoma cell lines OACP4C or OACM5.1C. In addition, copy number alterations of chromosomes were increased in CP-A but not in OACP4C or OACM5.1C, suggesting that acid exposure is involved in cancer development by induction of chromosomal instability rather than in cancer progression during esophageal carcinogenesis. Alvarez et al. identified widespread hypomethylation occurring as an early event in the progression of BE^51^, which supports our data suggesting that DNA hypomethylation is involved in cancer development in CP-A.

The expression levels of SAT increased in concert with the extent of α-Sat DNA hypomethylation on exposure to acid and bile acid, which was recognized in CP-A but not in OACP4C or OACM5.1C. Based on our previous study showing that overexpression of SAT induced a change in copy number at specific chromosomes^30^, we investigated its relationship to copy number alterations in CP-A, OACP4C and OACM5.1C using CGH analysis. CGH analysis demonstrated that copy number alterations at specific chromosomes were seen in CP-A, where expression levels of SAT were increased, whereas no alterations were recognized in OACP4C or OACM5.1C, where expression levels of SAT remained unchanged. Changes in copy number were induced in concert with the extent of DNA hypomethylation of α-Sat at the centromere region. These data implied that exposure to acid and bile acid led to CIN in CP-A by the induction of DNA hypomethylation of α-Sat followed by aberrant expression of SAT. CP-A exhibited considerable changes in morphology and an increased number of cells with abnormal segregation after three exposures to acid and bile acid. Our experiments revealed changes in morphology and copy number alterations of chromosomes by exposing a naïve Barrett’s epithelium cell line to short-term exposure to acid and bile acid.

The exposure of acid and bile acid may affect gene expression through DNA methylation at the promoter region. We identified several downregulated genes located in chromosomes 5 and 9 of the CP-A cells after exposure to acid and bile acid, where the loss of chromosome was determined using CGH analysis, but DNA methylation was not altered. In addition, some of the genes were involved in cell cycle and cell division pathways (Supplementary Table S2), which may contribute to the development of cancer in BE in concert with CIN.

In the tissue specimens of 14 patients who underwent gastrectomy for esophagogastric cancer, no major correlation was found between clinicopathological features and α-Sat RDL of tissue specimens. To explore whether acid-induced hypomethylation is involved in the development of Barrett's adenocarcinoma in clinical specimens, the relative hypomethylation levels of α-Sat was compared between tumor tissues and squamous epitheliums in 14 patients with Barrett's adenocarcinoma. The α-Sat RDL of tumor tissues was higher than that of squamous epitheliums, suggesting acid-induced DNA hypomethylation may be involved in the process of carcinogenesis in patients with Barrett's esophageal adenocarcinoma.

In conclusion, we demonstrated for the first time, that exposure to gastric and bile acids enhanced DNA hypomethylation in the centromere region and led to CIN in Barrett’s epithelial cell line. We believe that our findings could be instrumental in highlighting the significance of DNA hypomethylation in interpreting the direct association between extensive exposure to acid and bile acids and CIN, which could be involved in a potential mechanism underlying the development of EGJ carcinoma originating from BE.

## Materials and methods

### Patients and tissue specimens

Tumor tissue samples were obtained from 14 patients who underwent gastrectomy for Barrett's esophageal adenocarcinoma between January 2014 to February 2021 at the Saitama Medical Center, Jichi Medical University, Saitama, Japan. Patient characteristics and pathological findings are presented in Supplementary Table S3. In this study, Barrett's esophageal adenocarcinoma was endoscopically and histopathologically proven. Adenocarcinoma and squamous mucosal tissues were collected from formalin-fixed paraffin-embedded (FFPE) tissue specimens by the macrodissection method. All patients provided written informed consent to examine their tissue and the use of their clinical data. The study protocol was approved by the research ethics committee of Jichi Medical University and conformed to the ethical guidelines of the World Medical Association Declaration of Helsinki.

### Cell lines

Three different cell lines were used in this study. Two human esophageal adenocarcinoma cell lines, OACP4C (ATCC, Manassas, VA, USA), and OACM5.1C (ECACC, Salisbury, UK) were maintained in RPMI-1640 medium supplemented with 10% heat-inactivated fetal bovine serum. A BE metaplastic cell line that was immortalized by stable transfection of the human telomerase reverse transcriptase (TERT), CP-A (ECACC, Salisbury, UK), was maintained in MCDB-153 medium with supplementation that included 5% fetal bovine serum, 20 ng/mL recombinant human epidermal growth factor (EGF), 140 μg/mL bovine pituitary extract (BPE), 0.4 μg/mL hydrocortisone, 20 mg/L adenine, 1 × ITS supplement, 4 mM glutamine, and 8.4 μg/mL cholera toxin.

### Cell line treatment with acids and bile acids

At 80% confluence, cells were treated at several pHs, such as pH 7.2, 6.8, 6.4, 6.0, 5.0, 4.0, and a combination of 100 μm DCA for 48 h. The pH was adjusted using hydrochloric acid. Cells were then harvested using 0.05% trypsin solution and cultured in the basal medium. When the cells achieved 80% confluence again, they were exposed to the medium under the same acidic conditions. When the cells reached subconfluent cultures after a total of two exposures for OACP4C, OACM5.1C and three exposures for CP-A, they were collected and used in the following assays.

### Cell viability

Cell viability tests were performed using 3-(4,5-Dimethylthiazol-2-yl)-2,5-Diphenyltetrazolium bromide (MTT; Sigma-Aldrich, USA). Cells were seeded in 96-well plates (10,000 cells/well) for 24 h and then exposed to a medium containing acid and bile acid for 1 h. The following procedure was performed, according to the manufacturer’s protocol: At the end of treatment, 10 µL MTT solution was added to each well, and after incubation for 2 h, 100 µL MTT solubilizing buffer was added to each well. Absorbance was measured at 570 nm and 690 nm (measured as a reference) using a microplate reader (Thermo Fisher Scientific, Inc., Waltham, MA, USA). Cell viability was calculated using the following formula:

$$ \% {\text{ cell viability}} = {\text{OD Sample }}/{\text{OD Control}} \times {1}00 $$

### DNA extraction and bisulfite modification

Genomic DNA of cell lines was isolated and purified using an EZ1 DNA Tissue Kit (Qiagen, Hilden, Germany) according to the manufacturer's instructions. DNA purity was assessed by spectrophotometry (Nanodrop, ND1000; PeqLab, Erlangen, Germany) in the absence of bands at 260 and 280 nm, and all 260/280 ratios were confirmed to be in the range of 1.8–2.0. DNA samples were used in the following processes: MethyLight Methods, array CGH, and methylation microarrays.

Bisulfite treatment was used to chemically modify genomic DNA using sodium bisulfite. In this treatment, cytosine is deaminated and converted to uracil, whereas methylated cytosine, 5-methylated cytosine (5-mC), remains unaffected by the treatment. When PCR was performed on a sample of bisulfite-treated DNA, the amplified DNA sequence revealed sequence differences between the methylated and unmethylated DNA. In this study, sodium bisulfite conversion of genomic DNA was performed using an EpiTect Plus DNA Bisulfite Kit (QIAGEN). DNA (1 µg in up to 40 µL), was processed according to the manufacturer’s instructions. Genomic DNA of clinical specimens was extracted from FFPE tissue samples using EpiTect® Fast Bisulfite Conversion Kits (QIAGEN) and treated with Bisulfite according to the protocol.

### MethyLight methods

After bisulfite modification, each sample was examined using the MethyLight assay for LINE-1 and α-Sat sequences^52^. Two sets of primers and probes specifically designed to bind to bisulfite-converted DNA were used in the reaction: one set of LINE-1 and α-Sat primers and a probe for methylated or unmethylated target analyses, and another set of primers for the reference locus, ALU-C, as previously reported^52^. The primer sequences are as follows: Satellite alpha F 5ʹ-TTG ATG GAG TAT TTT TAA AAT ATA TGT TTT GTA GT-3ʹ, R 5-AAA TTC TAA AAA TAT TCC TCT TCA ATT ACA TAA A-3ʹ, Probe 5ʹ-FAM-TTT ATC CCA TTT CCA ACA AA-MGB-3ʹ, LINE-1 F 5ʹ-TTT ATT AGG GAG TGT TAG ATA GTG GGT G-3ʹ, R 5ʹ-TTT ATT AGG GAG TGT TAG ATA GTG GGT G-3ʹ, Probe 5ʹ-FAM-CAC CCT ACT TCA ACT CAT ACA CAA TAC ACA CAC CC-BHQ-3ʹ, Alu-C4 F 5ʹ-GGT TAG GTA TAG TGG TTT ATA TTT GTA ATT TTA GTA-3ʹ, R 5ʹ-ATT AAC TAA ACT AAT CTT AAA CTC CTA ACC TCA-3ʹ, Probe 5ʹ-FAM-CCT ACC TTA ACC TCC C-MGB-3ʹ. MethyLight data are reported as a relative level between the values derived from the real-time PCR standard curve and plotted as log (quantity) vs. threshold cycle (Ct) values for the unmethylated reaction as well as for a methylation-independent reaction.

In the whole-genome amplification method, completely unmethylated DNA from human genomic DNA (Promega, Madison, USA) was used as a reference to calculate the relative hypomethylation levels. In the whole-genome amplification method, fully unmethylated DNA from human genomic DNA was used as a standard to calculate relative hypomethylation levels. The relative demethylation level (RDL) was defined as (LINE-1 or α-Sat reaction/ALU-C reaction) sample/(LINE-1 or α-Sat reaction/ALU-C reaction) fully unmethylated control DNA, as previously reported^30,32,52^. Thermal cycling was initiated with a denaturation step at 95 °C for 10 s, followed by 50 cycles at 95 °C for 5 s and 60 °C for 30 s. PCR was performed on a QuantStudio 12 K Flex Real-Time PCR system (Applied Biosystems, Carlsbad, CA, USA) with a final reaction volume of 20 µL containing Premix Ex Taq (Takara Bio Inc., Otsu, Japan), 200 nM of each primer, and 200 nM probe. For each MethyLight reaction, 1 µL of bisulfite-modified DNA solution was used.

### RNA extraction and reverse transcription-quantitative polymerase chain reaction assays for RNA transcript expression

To entrain the cells to the mitotic phase, subconfluent cells were exposed to a medium containing 300 ng/mL nocodazole and incubated for 25 h. After a 40-min rest in a normal medium, total RNA was extracted from the cultured cells using the RNeasy Mini kit (QIAGEN) according to the manufacturer's instructions. We confirmed that the A260/A280 ratio was approximately 2.0, and the A260/230 ratio was over 2.0. After reverse transcription (RT) using the High Capacity RNA-to-cDNA kit (Applied Biosystems, Carlsbad, CA, USA), RT-qPCR assays were performed using TB Green Premix Ex Taq (Takara Bio, Otsu, Japan) and the QuantStudio 12 K Flex Real-Time PCR system (Applied Biosystems). The thermal cycling conditions were as follows: denaturation at 95 °C for 30 s; 40 cycles of annealing at 95 °C for 5 s and 60 °C for 30 s, and extension at 95 °C for 15 s, 60 °C for 60 s, and 95 °C for 15 s. The primer sequences are as follows: Satellite alpha Transcripts F 5ʹ-AAG GTC AAT GGC AGA AAA GAA -3ʹ, R 5ʹ-CAA CGA AGG CCA CAA GAT GTC-3ʹ, Glyceraldehyde-3-phosphate dehydrogenase (GAPDH) as the internal control F 5ʹ-CCA TCA TGA AGT GTG ACG TGG-3ʹ, R 5ʹ-GTC CGC CTA GAA GCA TTT GCG-3ʹ, ADAM19 F 5ʹ-GCC ATA GAT TCC TTT CAG CC-3ʹ, R 5ʹ-ATT CAC ATT GCC CTG GAT CC-3ʹ, CENPH F 5ʹ-TGC AAG AAA AGC AAA TCG AA-3ʹ, R 5ʹ-ATC CCA AGA TTC CTG CTG TG-3ʹ, DPYSL3 F 5ʹ-GGT CCC GCG GCA GAA ATA C-3ʹ, R 5ʹ-GGC ATC GAA ATC CAG CGT CT-3ʹ, ARHGEF39 F 5ʹ-GGA TCC TGA AAG CCA AGG GG-3ʹ, R 5ʹ-TCC AGG TAG GGA AGC AGC TC-3ʹ.

### Microarray-based comparative genomic hybridization (array CGH)

Array CGH was performed using the SurePrint G3 Human CGH Microarray Kit 8 × 60 k (Agilent Technologies Inc., Palo Alto, CA, USA). Labeling and hybridization were performed using the SureTag DNA Labeling kit and Oligo aCGH/ChiP‑on‑chip Hybridization kit (Agilent Technologies Inc.), according to the manufacturer’s protocol (Protocol v8.0). Genomic DNA derived from each cell line without acid or DCA was compared to a Human Male Reference DNA (Agilent). Genomic DNA of each cell line without acid or DCA was labeled with Cy5-dUTP and compared to a reference DNA, which was labeled with Cy3-dUTP (i.e., naïve CP-A vs reference). Genomic DNA of each cell line treated with acid and DCA was labeled with Cy5-dUTP and compared to that of corresponding cell line treated without acid or DCA, which was labeled with Cy3-dUTP (i.e., CP-A treated with acid and DCA vs naïve CP-A). After washing the array slides, they were scanned using an Agilent Technologies Microarray scanner, and the resulting data were analyzed using the Agilent Cytogenomics software program v.5.1. (Agilent Technologies Inc., Palo Alto, CA, USA).

### Immunocytochemistry

Immunocytochemistry was performed, as previously reported^30^. Cells were cultured on cover glasses in 24 well plates. After treatment with 300 ng/mL nocodazole for 24 h, the cells were fixed in 4% paraformaldehyde/PBS at room temperature for 10 min and then washed three times with PBS. The cells were permeabilized with 0.2% Triton X-100 (Agilent Technologies Inc.) at room temperature for 10 min and blocked with 10% normal goat serum (Thermo Fisher Scientific, Inc.) at room temperature for 30 min. The cells were washed three times with PBS and incubated with anti-α-tubulin (#ab52866; Abcam, Cambridge, UK; 1:500 for OACP4C and 1:1000 for CP-A) at 4 °C overnight. Following extensive washing with PBS, the samples were incubated with Alexa-594-conjugated antirabbit IgG secondary antibody (#ab150080; Abcam; 1:200 dilution for OACP4C and 1:500 dilution for CP-A) at room temperature for 60 min, and then Hoechst 33342 (Thermo Fisher Scientific, Inc.; 1 µg/mL) was added for nuclear staining and for an additional 15 min. The cover glasses were washed three times with PBS, mounted in ProLong Diamond Antifade Mountant (Thermo Fisher Scientific, Inc.), and sealed with nail polish. Images were acquired using a BZ-X700 fluorescence microscope (Keyence, Osaka, Japan).

### mRNA gene expression arrays

Gene expression arrays were performed to compare gene expression differences between the control and pH 6.4 and DCA-exposed groups in CP-A. After extraction of RNA, RNA concentration and integrity were assessed with microfluidic analysis using the Agilent 2100 Bioanalyzer and denaturing agarose gel electrophoresis. Labeling and hybridization were performed as follows: 100 ng of total RNA was amplified and labeled using the WT Plus reagent Kit (Affymetrix) and then hybridized to Clariom S human (Affymetrix, USA). Washing and scanning were conducted using the GeneChip System of Affymetrix (GeneChip Hybridization Oven 645, Gene-Chip Fluidics Station 450, GeneChip Scanner 3000 7G). A TA threshold of FC ≤ -2 for downregulated genes and FC ≥ 2 for upregulated genes was used. The list of genes was analyzed using Transcriptome Analysis Console (TAC). A series of processes and microarray analyses were performed at GeneticLab Co. Ltd. (Hokkaido, Japan).

### Methylation microarrays

Genomic DNA was extracted using a previously described method. DNA quality was assessed using Nanodrop and agarose gel electrophoresis. gDNA (100 ng) was digested with TaqaI (NEB R0149) at 65 °C for 2 h followed by MspI (NEB R0106) at 37 °C overnight. Following enzymatic digestion, samples were used for library generation using the Ovation RRBS Methyl-Seq System (Tecan 0353-32) following the manufacturer’s instructions. Digested DNA was randomly ligated, and following fragment end repair, bisulfite conversion using the EpiTect Fast DNA Bisulfite Kit (Qiagen 59824) was performed following the Qiagen protocol. After conversion and clean-up, the samples were amplified using the Ovation RRBS Methyl-Seq System protocol for library amplification and purification. Libraries were analyzed using the Agilent 2200 TapeStation System and quantified using the KAPA Library Quant Kit ABI Prism qPCR Mix (Roche KK4835). Libraries were sequenced using the NextSeq 550 at SE75. A series of processes and microarray analyses were performed at Active Motif Co. Ltd (CA, USA). Promoter coverages and methylated fractions were determined by Integrative Genomics Viewer (IGV).

### Statistical analyses

All statistical analyses were performed using JMP^®^ 11 (SAS Institute Inc., Cary, NC, USA). Continuous variations are expressed as the mean ± standard error. When necessary, differences in qualitative variables were evaluated using Welch’s *t*-test or Fisher's exact test. Continuous variables were compared using analysis of variance (ANOVA) with a post-hoc test and Student's *t*-test. The non-parametric Mann–Whitney U test was used for those variables that did not follow a normal distribution. Differences in promoter coverages and methylated fractions determined in methylation micro arrays were evaluated using Fisher's exact test. All reported P-values were two-sided, and P-values < 0.05 were considered to represent a statistically significant result.

## Supplementary Information

Supplementary Information 1.

Supplementary Information 2.

Supplementary Information 3.

Supplementary Information 4.

Supplementary Information 5.

Supplementary Information 6.

## Data Availability

Microarray data generated during the current study are available in the Gene Expression Omnibus (GEO) database with the accession number GSE217263.

## References

[1] Ferlay J (2021). Cancer statistics for the year 2020: An overview. Int. J. Cancer..

[2] Tahara T, Arisawa T (2015). DNA methylation as a molecular biomarker in gastric cancer. Epigenomics.

[3] Maekita T (2006). High levels of aberrant DNA methylation in Helicobacter pylori-infected gastric mucosae and its possible association with gastric cancer risk. Clin. Cancer Res..

[4] Fukase K (2008). Effect of eradication of Helicobacter pylori on incidence of metachronous gastric carcinoma after endoscopic resection of early gastric cancer: An open-label, randomised controlled trial. The Lancet.

[5] Wang C (2017). Changing trends in the prevalence of H. pylori infection in Japan (1908–2003): A systematic review and meta-regression analysis of 170,752 individuals. Sci. Rep..

[6] He H (2020). Trends in the incidence and survival of patients with esophageal cancer: A SEER database analysis. Thorac. Cancer.

[7] Honda M (2017). Long-term trends in primary sites of gastric adenocarcinoma in Japan and the United States. J. Cancer.

[8] Westra WM (2018). Smokeless tobacco and cigar and/or pipe are risk factors for Barrett Esophagus in male patients with gastroesophageal reflux disease. Mayo Clin. Proc..

[9] Kubo A (2013). Sex-specific associations between body mass index, waist circumference and the risk of Barrett's oesophagus: A pooled analysis from the international BEACON consortium. Gut.

[10] Singh S (2013). Central adiposity is associated with increased risk of esophageal inflammation, metaplasia, and adenocarcinoma: A systematic review and meta-analysis. Clin. Gastroenterol. Hepatol..

[11] Cook MB, Wild CP, Forman D (2005). A systematic review and meta-analysis of the sex ratio for Barrett's esophagus, erosive reflux disease, and nonerosive reflux disease. Am. J. Epidemiol..

[12] Argyrou A (2018). Risk factors for gastroesophageal reflux disease and analysis of genetic contributors. World J. Clin. Cases.

[13] Peters Y (2019). Barrett oesophagus. Nat. Rev. Dis. Primers.

[14] Olsen CM (2011). Population attributable fractions of adenocarcinoma of the esophagus and gastroesophageal junction. Am. J. Epidemiol..

[15] Dvorak K (2007). Bile acids in combination with low pH induce oxidative stress and oxidative DNA damage: Relevance to the pathogenesis of Barrett's oesophagus. Gut.

[16] Tselepis C (2003). Upregulation of the oncogene c-myc in Barrett's adenocarcinoma: Induction of c-myc by acidified bile acid in vitro. Gut.

[17] Souza RF, Krishnan K, Spechler SJ (2008). Acid, bile, and CDX: The ABCs of making Barrett's metaplasia. Am. J. Physiol. Gastrointest. Liver Physiol..

[18] Tselepis C, Perry I, Jankowski J (2000). Barrett's esophagus: Disregulation of cell cycling and intercellular adhesion in the metaplasia-dysplasia-carcinoma sequence. Digestion.

[19] Nehra D, Howell P, Pye JK, Beynon J (1998). Assessment of combined bile acid and pH profiles using an automated sampling device in gastro-oesophageal reflux disease. Br. J. Surg..

[20] Suzuki H, Toyota M, Kondo Y, Shinomura Y (2009). Inflammation-related aberrant patterns of DNA methylation: detection and role in epigenetic deregulation of cancer cell transcriptome. Methods Mol. Biol..

[21] Klump B, Hsieh CJ, Holzmann K, Gregor M, Porschen R (1998). Hypermethylation of the CDKN2/p16 promoter during neoplastic progression in Barrett's esophagus. Gastroenterology.

[22] Emmett RA, Davidson KL, Gould NJ, Arasaradnam RP (2017). DNA methylation patterns in ulcerative colitis-associated cancer: A systematic review. Epigenomics.

[23] Hong J (2010). Acid-induced p16 hypermethylation contributes to development of esophageal adenocarcinoma via activation of NADPH oxidase NOX5-S. Am. J. Physiol. Gastrointest. Liver Physiol..

[24] Wang JS (2009). DNA promoter hypermethylation of p16 and APC predicts neoplastic progression in Barrett's esophagus. Am. J. Gastroenterol..

[25] Kaz AM, Grady WM, Stachler MD, Bass AJ (2015). Genetic and epigenetic alterations in Barrett's esophagus and esophageal adenocarcinoma. Gastroenterol. Clin. N. Am..

[26] Lai LA (2010). Deletion at fragile sites is a common and early event in Barrett's esophagus. Mol. Cancer Res..

[27] Salem ME (2018). Comparative molecular analyses of esophageal squamous cell carcinoma, esophageal adenocarcinoma, and gastric adenocarcinoma. Oncologist.

[28] Eden A, Gaudet F, Waghmare A, Jaenisch R (2003). Chromosomal instability and tumors promoted by DNA hypomethylation. Science.

[29] Gaudet F, Eden A, Jackson-Grusby L, Dausman J, Gray JW, Leonhardt H, Jaenisch R (2003). Induction of tumors in mice by genomic hypomethylation. Science.

[30] Ichida K (2018). Overexpression of satellite alpha transcripts leads to chromosomal instability via segregation errors at specific chromosomes. Int. J. Oncol..

[31] Kakizawa N (2019). High relative levels of satellite alpha transcripts predict increased risk of bilateral breast cancer and multiple primary cancer in patients with breast cancer and lacking BRCArelated clinical features. Oncol. Rep..

[32] Saito M (2012). The accumulation of DNA demethylation in Sat alpha in normal gastric tissues with Helicobacter pylori infection renders susceptibility to gastric cancer in some individuals. Oncol. Rep..

[33] White SA, Allshire RC (2004). Loss of Dicer fowls up centromeres. Nat. Cell Biol..

[34] Ting DT (2011). Aberrant overexpression of satellite repeats in pancreatic and other epithelial cancers. Science.

[35] Zhuang Z (1996). Barrett's esophagus: metaplastic cells with loss of heterozygosity at the APC gene locus are clonal precursors to invasive adenocarcinoma. Cancer Res..

[36] Walch AK (2000). Chromosomal imbalances in Barrett's adenocarcinoma and the metaplasia-dysplasia-carcinoma sequence. Am. J. Pathol..

[37] Cancer Genome Atlas Research, N. Comprehensive molecular characterization of gastric adenocarcinoma. *Nature* **513**, 202–209 10.1038/nature13480 (2014). 10.1038/nature13480 PMC4170219 25079317

[38] Nehra D, Howell P, Williams CP, Pye JK, Beynon J (1999). Toxic bile acids in gastro-oesophageal reflux disease: Influence of gastric acidity. Gut.

[39] Kauer WK, Stein HJ (2010). Emerging concepts of bile reflux in the constellation of gastroesophageal reflux disease. J. Gastrointest. Surg..

[40] Harmon JW, Doong T, Gadacz TR (1978). Bile acids are not equally damaging to the gastric mucosa. Surgery.

[41] Jenkins GJ (2007). Deoxycholic acid at neutral and acid pH, is genotoxic to oesophageal cells through the induction of ROS: The potential role of anti-oxidants in Barrett's oesophagus. Carcinogenesis.

[42] Bus P, Siersema PD, van Baal JW (2012). Cell culture models for studying the development of Barrett's esophagus: A systematic review. Cell Oncol. (Dordr).

[43] Coenraad M (1998). Is Barrett's esophagus characterized by more pronounced acid reflux than severe esophagitis?. Am. J. Gastroenterol..

[44] Savarino E (2010). Characteristics of gastro-esophageal reflux episodes in Barrett's esophagus, erosive esophagitis and healthy volunteers. Neurogastroenterol. Motil..

[45] Wong DJ, Barrett MT, Stoger R, Emond MJ, Reid BJ (1997). p16INK4a promoter is hypermethylated at a high frequency in esophageal adenocarcinomas. Cancer Res..

[46] Wong DJ (2001). p16(INK4a) lesions are common, early abnormalities that undergo clonal expansion in Barrett's metaplastic epithelium. Cancer Res..

[47] Bajpai M (2013). High resolution integrative analysis reveals widespread genetic and epigenetic changes after chronic in-vitro acid and bile exposure in Barrett's epithelium cells. Genes Chromosomes Cancer.

[48] Suzuki K (2003). The genomic damage estimated by arbitrarily primed PCR DNA fingerprinting is useful for the prognosis of gastric cancer. Gastroenterology.

[49] Suzuki K (2006). Global DNA demethylation in gastrointestinal cancer is age dependent and precedes genomic damage. Cancer Cell.

[50] Moyzis RK (1989). The distribution of interspersed repetitive DNA sequences in the human genome. Genomics.

[51] Alvarez H (2011). Widespread hypomethylation occurs early and synergizes with gene amplification during esophageal carcinogenesis. PLoS Genet..

[52] Weisenberger DJ (2005). Analysis of repetitive element DNA methylation by MethyLight. Nucleic Acids Res..

