# Supplementary figures and images for: Enhancement of DNA hypomethylation alterations by gastric and bile acids promotes chromosomal instability in Barrett’s epithelial cell line
Source: Sci Rep. 2022 Dec 1;12:20710. doi: 10.1038/s41598-022-25279-y (PMC9715700; doi:10.1038/s41598-022-25279-y)

**a**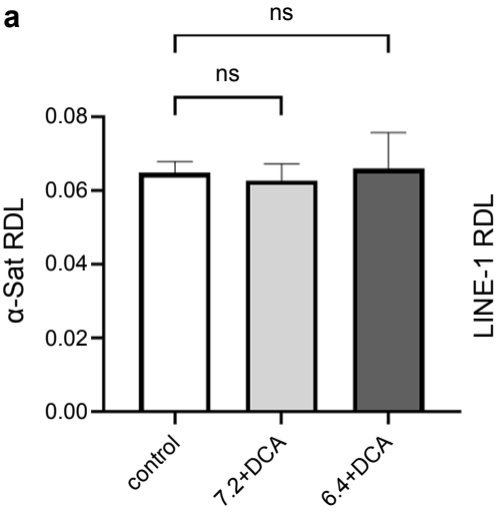**b**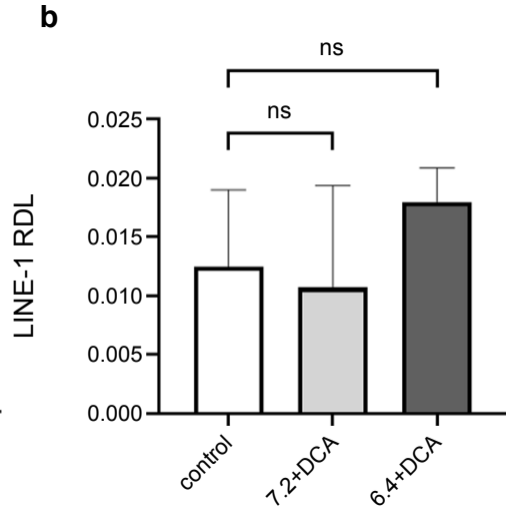**c**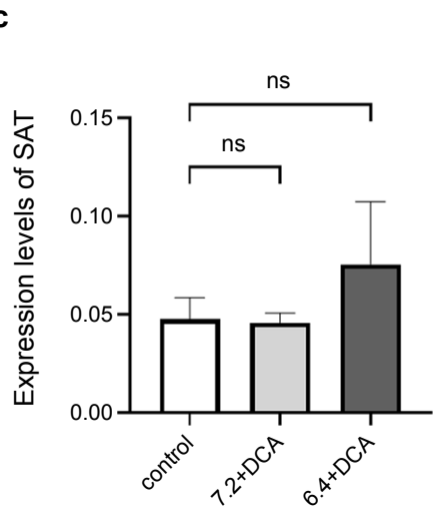

Supplement: Supplementary file 1 — Supplementary Information 1. [file 41598_2022_25279_MOESM1_ESM.pdf]

**a**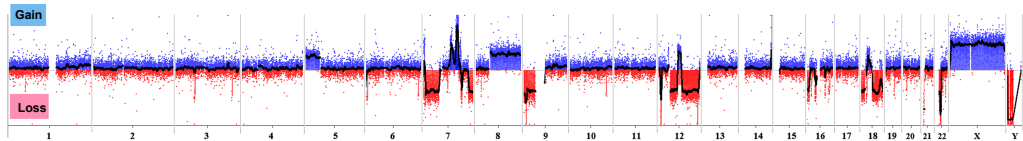**b**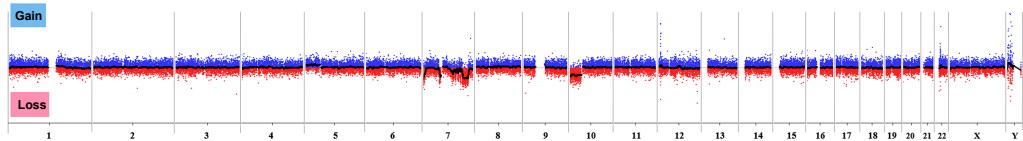**Chromosome**

Supplement: Supplementary file 2 — Supplementary Information 2. [file 41598_2022_25279_MOESM2_ESM.pdf]
