# Supplementary material for: Enhancement of DNA hypomethylation alterations by gastric and bile acids promotes chromosomal instability in Barrett’s epithelial cell line
Source: Sci Rep. 2022 Dec 1;12:20710. doi: 10.1038/s41598-022-25279-y (PMC9715700; doi:10.1038/s41598-022-25279-y)
Supplement: Supplementary file 3 — Supplementary Information 3. [file 41598_2022_25279_MOESM3_ESM.docx]

**Supplementary Table 1.** Results of gene annotation and pathway ontology of genes upregulated in CP-A treated with acid and DCA

| Term | Count | P-value | Benjamini |
| --- | --- | --- | --- |
| acute-phase response | 7 | 8.1E-4 | 3.8E-1 |
| Acute phase | 6 | 1.2E-4 | 4.5E-2 |
| cell chemotaxis | 6 | 3.9E-2 | 1 |
| Serum amyloid A protein | 4 | 9.1E-5 | 9.1E-2 |
| SAA | 4 | 1.2E-4 | 3.1E-2 |
| high-density lipoprotein particle | 4 | 2.4E-2 | 5.8E-1 |
| chemoattractant activity | 4 | 4.1E-2 | 1 |
| positive chemotaxis | 4 | 7.9E-2 | 1 |
| amyloid protein, SAA type | 3 | 2.7E-3 | 1.5E-1 |
| HDL | 3 | 7.7E-2 | 7.7E-1 |
| SAA: Serum amyloid A proteins  HDL: High Density Lipoprotein | | |  |
