# Supplementary material for: Enhancement of DNA hypomethylation alterations by gastric and bile acids promotes chromosomal instability in Barrett’s epithelial cell line
Source: Sci Rep. 2022 Dec 1;12:20710. doi: 10.1038/s41598-022-25279-y (PMC9715700; doi:10.1038/s41598-022-25279-y)
Supplement: Supplementary file 4 — Supplementary Information 4. [file 41598_2022_25279_MOESM4_ESM.docx]

**Supplementary Table 2.** Gene annotation and pathway ontology of genes down-regulated in CP-A treated with acid and DCA

| Term | Count | P-value | Benjamini |
| --- | --- | --- | --- |
| Cell cycle | 99 | 2.5E-29 | 3.5E-27 |
| Mitosis | 61 | 5.4E-28 | 5.8E-26 |
| cell division | 69 | 2.3E-24 | 2.0E-22 |
| cell division | 61 | 1.7E-19 | 2.3E-16 |
| mitotic nuclear division | 50 | 9.2E-19 | 8.3E-16 |
