# Supplementary material for: Enhancement of DNA hypomethylation alterations by gastric and bile acids promotes chromosomal instability in Barrett’s epithelial cell line
Source: Sci Rep. 2022 Dec 1;12:20710. doi: 10.1038/s41598-022-25279-y (PMC9715700; doi:10.1038/s41598-022-25279-y)
Supplement: Supplementary file 5 — Supplementary Information 5. [file 41598_2022_25279_MOESM5_ESM.docx]

| Factor | n=14 |
| --- | --- |
| Age | 73 (52-86) |
| Sex (male/female) | 12/2 |
| *H. Pylori* infection | 6 (42.8 %) |
| Differentiation (dif/undif) | 10/4 |
| Lymphatic invasion | 8 (57.1%) |
| Venous invasion | 9 (64.3%) |
| pStage (I/II/III/IV) | 8/5/0/1 |
| dif: Differentiated　　　undif: Undifferentiated | |

**Supplementary Table 3.** Clinicopathological features of the patients with EGJ cancer
