# Supplementary material for: Enhancement of DNA hypomethylation alterations by gastric and bile acids promotes chromosomal instability in Barrett’s epithelial cell line
Source: Sci Rep. 2022 Dec 1;12:20710. doi: 10.1038/s41598-022-25279-y (PMC9715700; doi:10.1038/s41598-022-25279-y)
Supplement: Supplementary file 6 — Supplementary Information 6. [file 41598_2022_25279_MOESM6_ESM.docx]

**Enhancement of DNA hypomethylation alterations by gastric and bile acids promotes chromosomal instability in Barrett’s epithelial cell line**

Iku Abe, Koichi Suzuki, Yasuaki Kimura, Sawako Tamaki, Yuhei Endo, Kosuke Ichida, Yuta Muto, Fumiaki Watanabe, Masaaki Saito, Fumio Konishi, Toshiki Rikiyama

**Supporting information**

**Supplementary Figure S1. Relative hypomethylation levels of alpha satellite and LINE-1 repetitive sequences and expression levels of satellite alpha transcripts when exposed to acid and deoxycholic acid in OACM5.1C**

To assess the relative hypomethylation levels, the relative demethylation levels (RDL) of alpha satellite (α-Sat) and LINE-1 repetitive sequences were determined. The expression of satellite alpha transcripts (SAT) was also calculated. There was no significant difference in α-Sat RDL, LINE-1 RDL and expression levels of SAT under several conditions of acid deoxycholic acid in OACM5.1C. ns; no significance

**Supplementary Figure S2. Copy number changes in OACM5.1C before and after the exposure to acid and deoxycholic acid**

Copy number changes were explored using microarray-based comparative genomic hybridization (array CGH). Representative copy number alterations identified in OACM5.1C before (a) and after (b) treatment with acid and DCA. Genomic DNA from cells without acid or DCA was labeled with Cy5-dUTP and compared to a Human Male Reference DNA (Agilent), labeled with Cy3-dUTP (naïve OACM5.1C vs reference). Genomic DNA from OACM5.1C treated with acid and DCA was labeled with Cy5-dUTP and compared to that of OACM5.1C treated without acid or DCA, which was labeled with Cy3-dUTP (OACM5.1C treated with acid and DCA vs naïve OACM5.1C). Change in copy number alterations before after treatment with acid and DCA were rarely seen in OACM5.1C (b). Gains are shown as blue lines in the upper area and losses are red lines in the lower area, respectively. Numbers represent the chromosome number. In each chromosome area, the short arm is located on the left side, and the long arm is on the right side.
